# Cystoscopic removal of a migrated intrauterine device to the bladder; a case report

**DOI:** 10.1186/s40834-019-0089-x

**Published:** 2019-07-01

**Authors:** Masnoureh Vahdat, Mansoureh Gorginzadeh, Ashraf Sadat Mousavi, Elaheh Afshari, Mohammad Ali Ghaed

**Affiliations:** 1 Rasoul Akram Hospital, Iran University of Medical Sciences (IUMS), Niayesh Ave, Sattarkhan St, Tehran, Iran; 2 Endometriosis Research Center, Rasoul Akram Hospital, Iran University of Medical Sciences (IUMS), Tehran, Iran; 3 Department of Urology, Rasoul Akram Hospital, Iran University of Medical Sciences (IUMS), Tehran, Iran

**Keywords:** Intrauterine device, Migration, Bladder, Cystoscopy

## Abstract

**Background:**

An intrauterine device (IUD) is a well-accepted means of reversible contraception. Migration of IUD to the bladder through partial or complete perforation has been rarely reported. This phenomenon could be strongly associated with history of prior cesarean sections (C-section) or early insertion of the device in the postpartum period.

**Case presentation:**

In this study, a case of copper IUD migration through cesarean scar defect is presented, in such a way that was successfully managed by cystoscopic removal. A 31-year-old female with a history of lower urinary symptoms referred to the clinic for her secondary infertility work-up. A copper IUD outside the uterus in the bladder was found using hysterosalpingraphy. A plain abdominal radiography also confirmed the presence of a T-shaped IUD in the pelvis. According to ultrasound, the copper IUD was partly in the bladder lumen and within the bladder wall. The patient had a history of an intrauterine device insertion eight years ago followingher second cesarean delivery. Three years later, her IUD was expelled, and another copper IUD was inserted. Thesecond copper IUD was alsoremoved while she decided to be pregnant. The patient finally underwent a hysteroscopic cystoscopy. The intrauterine device with its short arms embedded in the bladder wall was successfully extracted through the urethra.

**Conclusions:**

IUD insertion seems to be more challenging in women with prior uterine incisions and requires more attention. Cystoscopic removal should be considered as a safe and effective minimally invasive approach tomanage a migrated intrauterine device in the bladder.

## Introduction

IUD is still considered as a popular and cost-effective method of reversible contraception worldwide [1, 2]. A displaced or migrated IUD causes serious complications such as vesicouterine fistula, bowel perforation, hydronephrosis, and even renal failure [2–6]. Intravesical translocation of IUD is a rare phenomenon which could be associated with stone formation or bladder perforation [6–8]. Surgical removal either through endoscopy or open technique is the best recommended treatment [4–9]. We report the case of a reproductive-aged female whose migrated copper IUD was incidentally found through hysterosalpingography (HSG).

## Case presentation

**Fig. 1:**
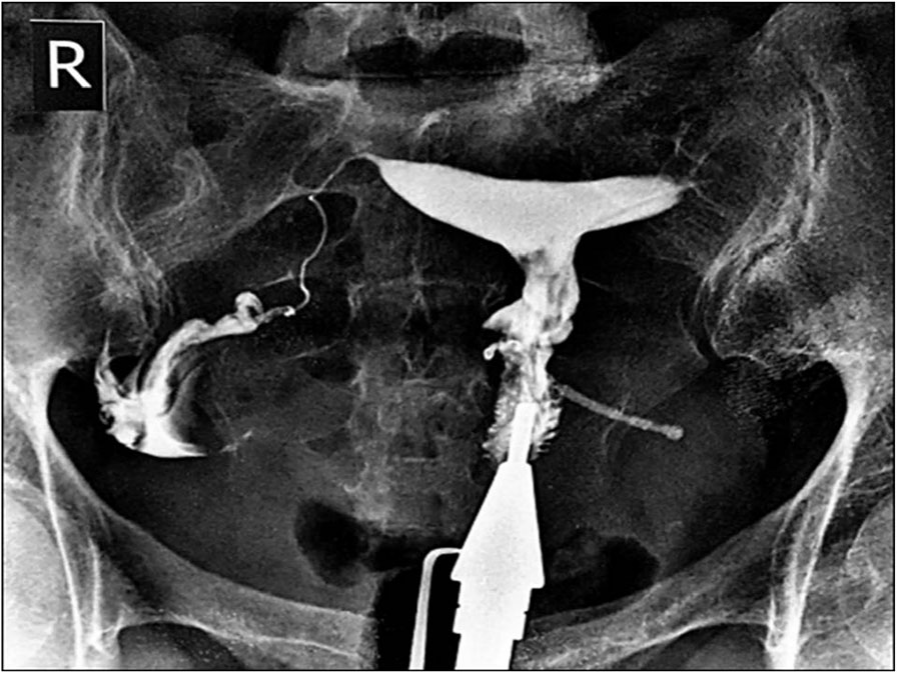
Hysterosalpingography demonstrating IUD out of uterine cavity

**Fig. 2:**
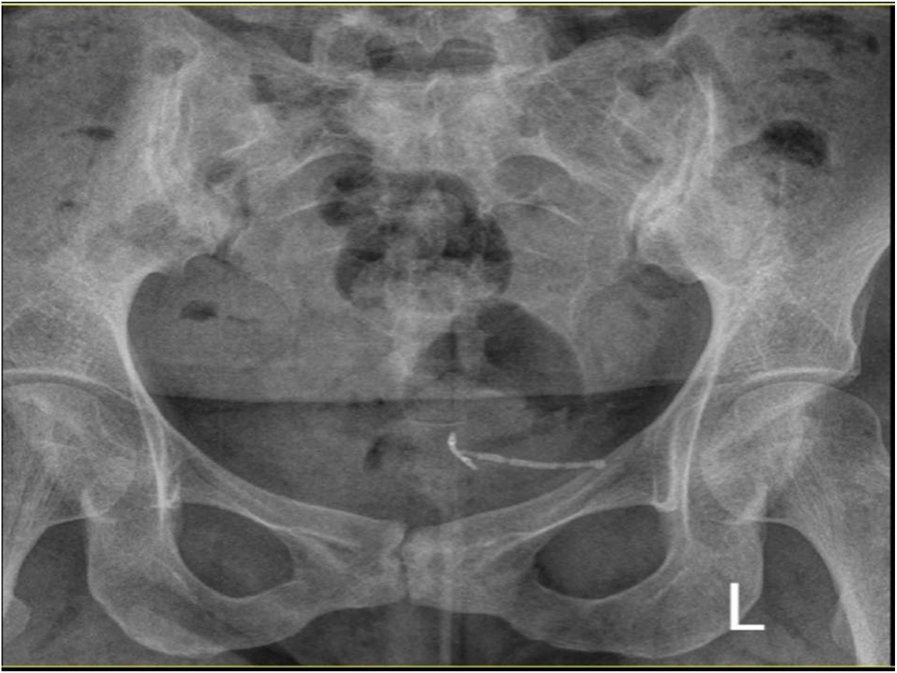
Abdominal X Ray revealing IUD

**Fig. 3:**
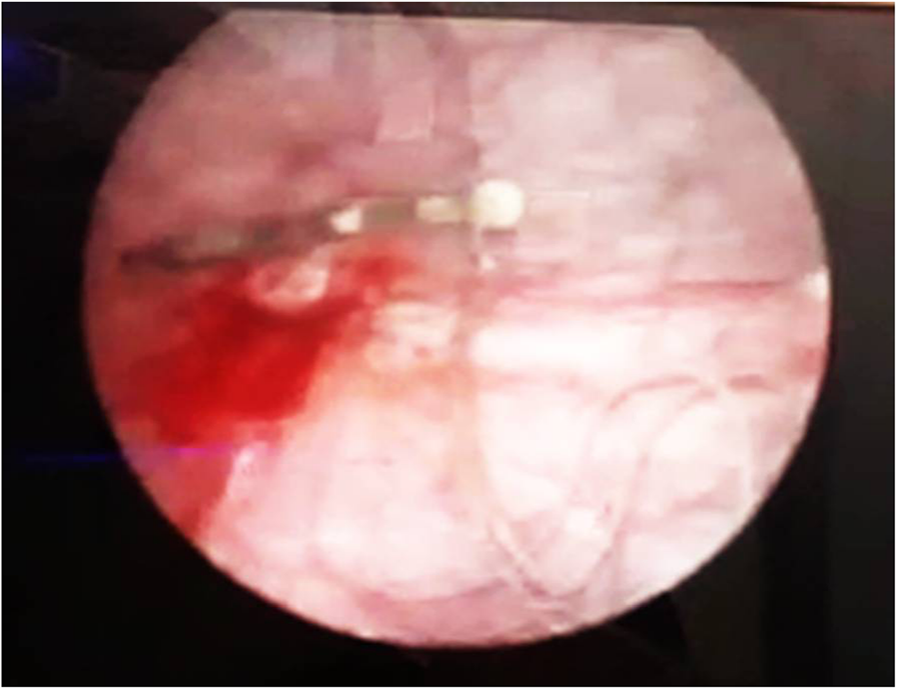
IUD with its strings in the bladder

**Fig. 4:**
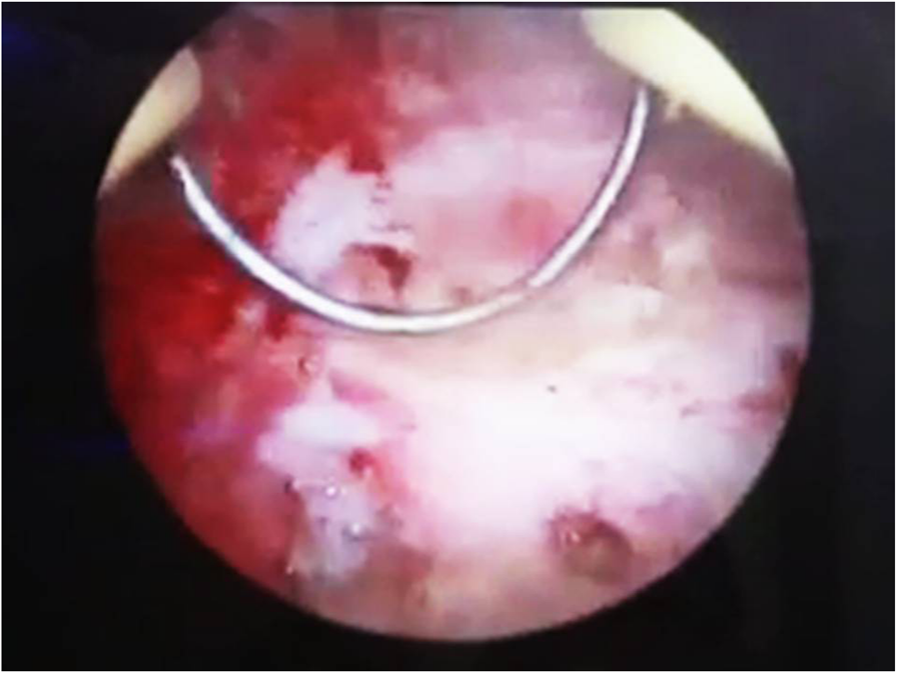
Two short arms embedded in the bladder wall

**Fig. 5:**
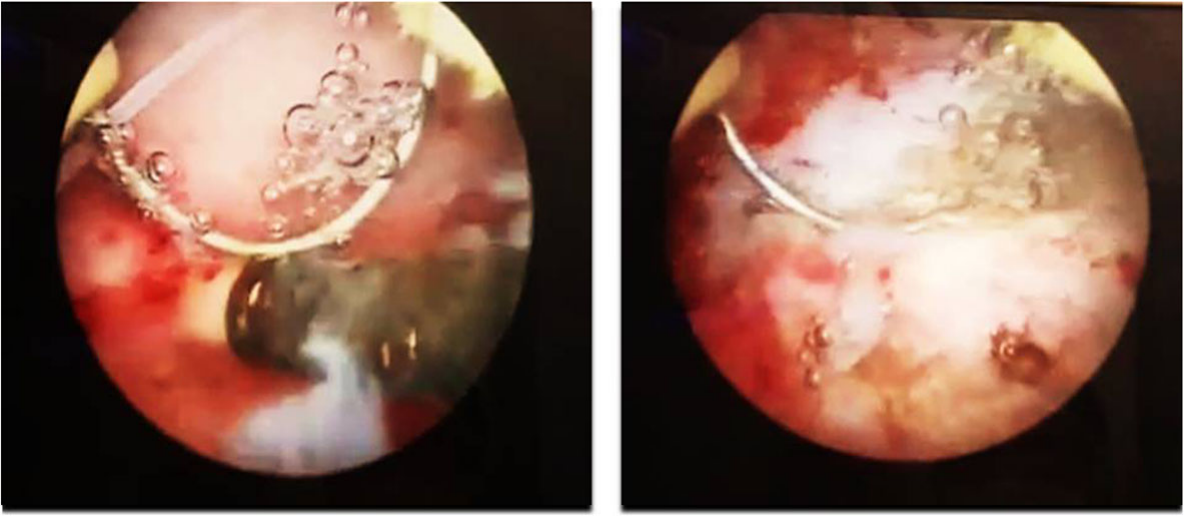
Loop electrode used for releasing the IUD

A 31-year-old female, gravida 2, para 2 (G2 P2), referred to the gynecologic clinic with a main complaint of secondary infertility during the last twelve months. Written informed consent was obtained from the patient for publication of this case report and any accompanying images. The patient had two previous C-sections. Her menstrual cycles were ovulatory. Spermogram was unremarkable and hormonal assay did not show any abnormality. HSG was performed and revealed a migrated copper IUD with its long tail out of the uterine cavity (Fig. 1). Plain abdominopelvic radiography also indicated a rotated T-shaped IUD in the pelvis (Fig. 2). A transvaginal ultrasound was also performed by a skilled radiologist who reported a copper IUD in the bladder lumen with a small portion of it within the bladder musculature. The patient had taken multiple courses of antibiotics for urinary tract infection (UTI), but her symptoms never disappeared. The patient also underwent cystoscopy for recurrent infection last year, but no pathological finding was detected. Eventually, the patient said that her symptoms were related to the possible adhesions following two previous operations. The patient had a history of IUD insertion following her last C-section about eight years ago. After three years, however, the patient decided to remove it due to recurrent vaginal secretions. Three years later, her IUD was expelled, and another copper IUD was inserted. The patient removed the other copper IUD for becoming pregnant about two years ago. The patient was very confident of its removal, but her recent HSG, interestingly, demonstrated a rotated copper IUD in the pelvic cavity. Physical examination was essentially normal. A baseline complete blood count, urea, and creatinine levels were normal. The patient was candidate for a hysteroscopic cystoscopy. Hysteroscopy was normal except for a small fibrotic defect at the lower segment of the uterus. During cystoscopy, the long tail of the copper IUD was found on the posterolateral border of the bladder far from ureteral offices (Fig. 3). The two short arms adhered to each other were embedded in the mucosal and muscular layer (Fig. 4). No calculus was observed within the bladder cavity. Using glycine as the media, a mono-polar loop entered the bladder. A gentle and brief cautery was applied on the mucosa where the shadow of a short arm of the device was observed. The copper IUD was safely removed through the urethra using a special grasper. There was no major defect in the place of the copper IUD in the bladder. The bleeding points were effectively cauterized (Fig. 5). The patient was discharged the next day with an indwelling catheter. After catheter removal, the patient did not complain of any urinary symptoms.

## Discussion

The current study presented the case of a multiparous female with two copper IUD insertions. The first insertion was right after her C-section. Indeed, the first copper IUD, mistakenly thought to have been expelled out of uterus through vagina, had migrated spontaneously from the uterine cavity to the bladder. It could have happened with movement of the device through the cesarean scar defect or after a uterine perforation as a result of improper insertion of the device. The exact mechanism of migration is unknown, but the fact that she had two prior C-sections justifies the first explanation [8–11].

The patient did not have any complaint of abnormal uterine bleeding except for persistent urinary symptoms wrongly treated with antibiotics. After her normal cystoscopy in another center without abnormal findings, the patient did not follow any further evaluation. The main cause of all symptoms was unexpectedly found using HSG. Among the factors increasing the chance of uterine perforation following IUD insertion such as unusual position of the uterus (acutely anteverted or retroverted), recent pregnancy and insertion by an inexperienced physician or midwife, special attention should be paid to the previous hysterotomy scars which could weaken the uterine wall. It seems that more careful follow-up after IUD insertion is required in women with multiple uterine scars. For this case, when the healthcare provider could not find the device in the cavity, further evaluation such as a simple abdominopelvic X-ray could be done. The possibility of spontaneous expulsion of the IUD should have been made only after thorough inspection of the abdomen and pelvis by different imaging methods. Intravesical migration of the IUD is not a new phenomenon and has been reported in many studies [4, 6–13].

According to potential severe complications of this phenomenon such as bladder or bowel perforation, it is important to confirm the proper place of the device in the uterine cavity as soon as possible [7–9]. Therefore, it is recommended to do regular exams to observe and palpate the strings of the IUD along the ultrasound immediately after insertion to affirm the correct insertion [12, 13]. The incidence of bladder perforation following IUD insertion is less than 5 per 1000 cases whose incidence can grow in cases of weakened uterine walls as in the current case [8, 9, 14, 15]. There were two other similar case reports by Niu et al. and Guner et al., in which IUD migration from previous cesarean scar defects happened and resulted in bladder perforation [16, 17].

Therefore, all health care professionals should be aware of the challenging aspect of IUD insertion particularly in women with prior uterine scars. Unlike the two mentioned studies, bladder perforation did not occur in the current case and the IUD was removed successfully through the urethra. The bleeding points at the site of the IUD removal were briefly and superficially cauterized without any complications. This migrated IUD had been misplaced since at least five years ago. Unlike some other studies in which the migrated IUD into the bladder was associated with stone formation [7–9], in the current case no stone was found in the bladder. Since the previous cystoscopy was normal in the current case, the migration to the bladder could have occurred recently or just after the first cystoscopy.

Surgical removal of the device is a definite treatment [18]. Both open and endoscopic techniques could be applied for this purpose. Initially minimally invasive approaches are adopted, while open surgical methods are undertaken only if they fail. For the current case, as with other studies [9, 13, 14], hysteroscopy and cystoscopy were sufficient for the management of this complication of the IUD insertion.

## Conclusion

IUD insertion seems to be more challenging in women with prior uterine incisions and requires more attention. Cystoscopic removal can be considered as a safe and effective minimally invasive approach to manage a migrated intrauterine device in the bladder.

## Abbreviations

C-section: Cesarean sections
HSG: Hysterosalpingography
IUD: Intrauterine device

## References

[1] Cleland K, Zhu H, Goldstuck N, Cheng L, Trussell J (2012). The efficacy of intrauterine devices for emergency contraception: a systematic review of 35 years of experience. Hum Reprod.

[2] Karsmakers R., Weis-Potters A. E., Buijs G., Joustra E. B. (2010). Chronic kidney disease after vesico-vaginal stone formation around a migrated intrauterine device. Case Reports.

[3] Wang L, Li Y, Zhao XP, Zhang WH, Bai W, He YG (2017). Hydronephrosis caused by intrauterine contraceptive device migration: three case reports with literature review. Clin Exp Obstet Gynecol..

[4] El-Hefnawy AS, El-Nahas AR, Osman Y, Bazeed MA (2008). Urinary complications of migrated intrauterine contraceptive device. Int Urogynecol J Pelvic Floor Dysfunct.

[5] Toumi O, Ammar H, Ghdira A, Chhaidar A, Trimech W, Gupta R (2018). Et al. pelvic abscess complicating sigmoid colon perforation by migrating intrauterine device: a case report and review of the literature. Int J Surg Case Rep.

[6] Madden A, Aslam A, Nusrat NB (2016). A case of migrating "Saf-T-coil" presenting with a vesicovaginal fistula and vesicovaginal calculus. Urol Case Rep.

[7] Shin DG, Kim TN, Lee W (2012). Intrauterine device embedded into the bladder wall with stone formation: laparoscopic removal is a minimally invasive alternative to open surgery. Int Urogynecol J.

[8] Jeje EA, Ojewola RW, Atoyebi OA (2012). Intravesical migration of a failed and forgotten intrauterine contraceptive device after 20 years of insertion – a case report. Nig Q J Hosp Med.

[9] Sano M, Nemoto K, Miura T, Suzuki Y (2017). Endoscopic treatment of intrauterine device migration into the bladder with stone formation. J Endourol Case Rep.

[10] Johri V, Vyas KC (2013). Misplaced intrauterine contraceptive devices: common errors; uncommon complications. J Clin Diagn Res.

[11] Rokhgireh S, Mehdizadehkashi A, Chaichian S, Vahdat M, Nazari L, Tajbakhsh B (2017). Simultaneous extraction of a retained surgical gauze from bladder and uterus 17 years after cesarean section: a rare case report. Iran Red Crescent Med J.

[12] Golightly E, Gebbie AE (2014). Low-lying or malpositioned intrauterine devices and systems. J Fam PlannReprod Health Care.

[13] Dias T, Abeykoon S, Kumarasiri S, Gunawardena C, Padeniya T, D'Antonio F (2015). Use of ultrasound in predicting success of intrauterine contraceptive device insertion immediately after delivery. Ultrasound Obstet Gynecol.

[14] Brar R, Doddi S, Ramasamy A, Sinha P (2010). A forgotten migrated intrauterine contraceptive device is not always innocent: a case report. Case Rep Med.

[15] Sharma A, Andankar M, Pathak H (2017). Intravesical migration of an intrauterine contraceptive device with secondary Calculus formation. Korean J Fam Med.

[16] Niu H, Qu Q, Yang X, Zhang L (2015). Partial perforation of the bladder by an intrauterine device in a pregnant woman: a case report. J Reprod Med.

[17] Guner B, Arikan O, Atis G, Canat L, Çaskurlu T (2013). Intravesical migration of an intrauterine device. Urol J.

[18] Adiyeke M, Sanci M, Karaca I, Gökçü M, Töz E, Ocal E (2015). Surgical management of intrauterine devices migrated towards intra-abdominal structures: 20-year experience of a tertiary center. Clin Exp Obstet Gynecol.

